# A model for co-expression pattern analysis of genes implicated in angiogenesis and tumour cell invasion in cervical cancer

**DOI:** 10.1038/sj.bjc.6600471

**Published:** 2002-08-27

**Authors:** P O Van Trappen, A Ryan, M Carroll, C Lecoeur, L Goff, V G Gyselman, B D Young, D G Lowe, M S Pepper, J H Shepherd, I J Jacobs

**Affiliations:** Department of Gynaecological Oncology, Cancer Research UK Translational Oncology Laboratory, John Vane Science Centre, St. Bartholomew's and the Royal London School of Medicine and Dentistry, Queen Mary University of London, Charterhouse Square, London EC1M 6BQ, UK; Department of Nuclear Medicine, St. Bartholomew's and the Royal London School of Medicine and Dentistry, Queen Mary University of London, London EC1A 7BE, UK; Genome Centre, St. Bartholomew's and the Royal London School of Medicine and Dentistry, Queen Mary University of London, Charterhouse Square, London EC1M 6BQ, UK; Cancer Research UK Medical Oncology Laboratory, St. Bartholomew's and the Royal London School of Medicine and Dentistry, Queen Mary University of London, Charterhouse Square, London EC1M 6BQ, UK; Academic Department of Surgery, St. Bartholomew's and the Royal London School of Medicine and Dentistry, Queen Mary University of London, London EC1A 7BE, UK; Academic Department of Histopathology, St. Bartholomew's and the Royal London School of Medicine and Dentistry, Queen Mary University of London, London EC1A 7BE, UK; Department of Morphology, University Medical Centre, Geneva, Switzerland

**Keywords:** cervical cancer, angiogenesis, tumour cell invasion, co-expression pattern analysis

## Abstract

To date, numerous genes have been identified which are involved in both tumour neovascularisation (angiogenesis) and tumour cell invasion, and most of them are also expressed to some extent under normal physiological conditions. However, little is known about how these genes co-express in these settings. This study was undertaken to quantitate mRNA levels in normal and malignant cervical tissues of nine selected genes (VEGF_121_, VEGF_165_, VEGF_189_, VEGF-C, eIF-4E, b-FGF, TSP-2, MMP-2 and MMP-9) implicated in the above processes using real-time quantitative RT–PCR. In addition, the Spearman's rank correlation was used to determine their co-expression patterns. The transcript levels for the different VEGF-A splice variants (VEGF_121_, VEGF_165_, VEGF_189_) were at least 10-fold higher in the cancer cases, with the highest levels in the primary tumours demonstrating lympho-vascular space involvement. The lymphangiogenic factor VEGF-C and MMP-9 were upregulated 130- and 80-fold respectively in cervical cancers. The highest levels of VEGF-C mRNA were found in the lymph-node positive group. The transcript levels for b-FGF were similar in normal cervical tissue and early-stage cervical cancer, however, higher levels were found in the cervical cancers with advanced stage disease. Comparing gene transcript levels between recurrent and non-recurrent cervical cancer patients revealed significant differences (*P*=0.038) in transcript levels for the angiogenesis inhibitor TSP-2, with the highest levels in non-recurrent cases. Co-expression pattern analysis in normal cervical tissue revealed highly significant co-expressions (*P*<0.0001) between TSP-2 and most other genes analysed (VEGF_121_, VEGF_165_, VEGF-C, b-FGF and MMP-2). In cervical cancer, TSP-2 appears only to be highly co-expressed with MMP-2 (*P*<0.0001). In contrast to normal cervical tissue, we found a highly significant co-expression (*P*<0.0001) between MMP-9 and VEGF_189_ in cervical cancer. The combined application of real-time quantitative RT–PCR and Spearman's rank correlation identifies gene transcripts which are simultaneously co-expressed. Our results revealed a significant co-expression between the angiogenesis inhibitor TSP-2 and most other genes analysed in normal cervical tissue. In cervical cancer, we found a strong upregulation of VEGF-C and MMP-9 mRNA, with a highly significant co-expression between MMP-9 and VEGF_189_.

*British Journal of Cancer* (2002) **87**, 537–544. doi:10.1038/sj.bjc.6600471 www.bjcancer.com

© 2002 Cancer Research UK

## 

During tumour progression, two phases can be recognised with regard to neovascularization: a prevascular and a vascular phase. The transition from one phase to the other is referred to as the ‘angiogenic switch’ (Folkman, 1971, 2000; Carmeliet and Jain, 2000; Hanahan and Folkman, 1996). The current working hypothesis is that the ‘switch’ involves either the induction of a positive regulator and/or the loss of a negative regulator. In the healthy adult organism, endothelial cell turnover is very low. This is thought to be due to the dominance of endogenous negative regulators, since positive regulators are frequently detected in adult tissues in which there is apparently no angiogenesis. Endogenous negative regulators identified to date include members of the thrombospondin (TSP) family, angiostatin and endostatin (Volpert *et al*, 1995; O'Reilly *et al*, 1994, 1997). Among the positive endogenous regulators of tumour angiogenesis are the vascular endothelial growth factor (VEGF) and fibroblast growth factor (FGF) families of cytokines.

Molecular cloning has revealed at least five different isoforms of VEGF which are generated from a single mRNA by alternative splicing, and which have significantly different biochemical features and biological effects (Ferrara *et al*, 1991; Houck *et al*, 1991; Veikkola and Alitalo, 1999; Yancopoulos *et al*, 2000). There is recent evidence in breast cancer patients that levels of VEGF-A mRNA correlate with mRNA levels of the translation initiation factor eIF-4E, implying co-regulation of these genes (Scott *et al*, 1998). In several cancer types (breast, head and neck and prostate cancer) eIF-4E causes increased translational efficiency of several oncogene transcripts (e.g. cyclin D1 and c-Myc) and growth factors (e.g. basic-FGF and VEGF), leading to overexpression of their products (de Benedetti and Harris, 1999). Recent studies have shown that the growth factor VEGF-C plays a crucial role in the growth of lymphatic vessels (lymphangiogenesis), an effect which is mediated in part through VEGFR-3 (Jeltsch *et al*, 1997; Lymboussaki *et al*, 1998).

Many biological processes, including angiogenesis and tumour cell invasion, require extracellular proteolysis for degradation of the extracellular matrix. This allows endothelial and tumour cells to migrate through tissue stroma and subsequently to enter and spread via the blood and/or lymphatic systems. The matrix metalloproteinases (MMPs), including MMP-2 and MMP-9 (gelatinase-A and -B), are amongst the best studied proteases in this respect. Enhanced mRNA and protein levels of both MMP-2 and MMP-9 have been detected in breast, colon and pancreatic cancer (Campo *et al*, 1992). A recent study in transgenic mice suggests that MMP-9 triggers the angiogenic switch during carcinogenesis (Bergers *et al*, 2000).

Until recently, studies on angiogenesis in several cancers have largely been limited to analysis of microvascular density (MVD). However, little information is available regarding the co-expression of genes implicated in angiogenesis in different tumour types. Both angiogenesis and lymphangiogenesis are likely to be key processes in disease progression in cervical malignancy, given the well documented pattern of direct spread through parametrial tissues, lymphatic spread to pelvic lymph nodes, and haematogenous dissemination to distant organs. We have recently shown that approximately 50% of early-stage cervical cancers shed tumour cells to the pelvic lymph nodes (van Trappen *et al*, 2001). The lymphangiogenic factor VEGF-C may be a key element in this process.

Recent technical advances have provided us with opportunities to assess levels of gene transcription and to identify patterns of co-expression. Although microarray technology can measure the expression of thousands of genes simultaneously, it is a semi-quantitative technique (Schena *et al*, 1995; Eisen *et al*, 1998; Duggan *et al*, 1999; Lander, 1999; Liotta and Petricoin, 2000). As the genes involved in tumour angiogenesis are also expressed in normal tissues, a real understanding of gene expression in carcinogenesis requires an accurate and fully quantitative technique. Real-time quantitative RT–PCR fulfils this requirement and has recently been used to accurately quantitate transcription levels of a variety of genes (Heid *et al*, 1996).

The aim in our study was to identify which genes are co-expressed in tumours in which angiogenesis and/or lymphangiogenesis are prominent, and to determine whether a different co-expression pattern exists between normal and malignant cervical tissue. Transcripts of the following nine genes were analysed: VEGF_121_, VEGF_165_, VEGF_189_, VEGF-C, eIF-4E, basic-FGF (b-FGF), TSP-2, MMP-2 and MMP-9.

## MATERIALS AND METHODS

### Patients and samples

The patient population consisted of 52 women of whom 34 had malignant and 18 normal cervical tissue. Ethical approval was obtained from the Local Research Ethics Committee of the East London and City Health Authority. During the period between January 1998 to March 2000, radical hysterectomy and pelvic lymphadenectomy were carried out in 29 cervical cancer patients with early-stage disease confined to the uterus. Five patients were treated with primary radiotherapy for advanced stage disease (stage IIB–III) during the same time period, and cervical biopsies were taken before radiotherapy. None of the cancer patients had undergone any previous treatment to the cervix. Tissue specimens were taken from the periphery of the tumour, as it is known that neovascular hot spots occur predominantly at tumour margins (Tokumo *et al*, 1998). One portion was snap frozen for RT–PCR analysis while a neighbouring portion was fixed in 10% formaldehyde for histological examination to determine whether the biopsy specimen contained >80% tumour tissue. The histological cell types of the 34 cervical cancers were assigned according to the World Health Organisation (WHO) classification: 23 were classified as squamous cell carcinoma, 8 as adenocarcinoma, 2 as clear cell carcinoma and 1 as carcinoid. Clinical staging was determined using the International Federation of Gynaecology and Obstetrics (FIGO) classification: 6 were stage IA2, 15 were staged as stage IB1, 6 were stage IB2, 2 were stage IIA, 3 were stage IIB, 2 were stage III (Table 1

**Table 1:**
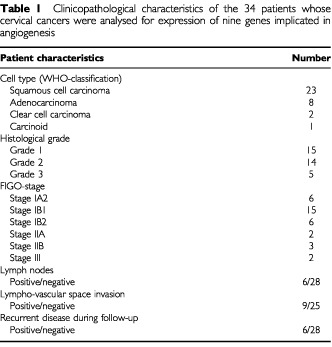
Clinicopathological characteristics of the 34 patients whose cervical cancers were analysed for expression of nine genes implicated in angiogenesis

: patient characteristics).

As controls, 18 biopsy specimens were obtained from normal cervices from patients who underwent a simple hysterectomy for non-malignant lesions in the corpus of the uterus. None of these patients had conditions, such as endometriosis, in which angiogenesis may be an important component. Light microscopic examination confirmed the normal histology.

### RNA extraction

Total RNA was extracted from all samples using the acid-guanidinium isothiocyanate-phenol-chloroform method as described previously (Chomczynski and Sacchi, 1987). Disintegration of the tissue was achieved by vortexing and by using a Dounce homogeniser. The RNA samples were stored in 50 μl diethyl pyrocarbonate (DEPC) treated distilled water at −80°C. RNAs were further purified using an RNeasy Mini Protocol for RNA Clean up (Qiagen Ltd., Crawley, UK) and quantitated in a Genequant spectrophotometer (Pharmacia, St Albans, UK).

### Primers and probes

Primers and probes were designed using Primer Express software (PE-ABI). The probes were designed specifically to span an intron in order to avoid potential amplification of small amounts of contaminating DNA in the analysed samples. The probes were labelled at the 5′ end with 6-carboxy fluorescein (FAM) and at the 3′ end with 6-carboxy-tetramethyl rhodamine (TAMRA). Primers and probes were stored at −20°C until use. Table 2

**Table 2:**
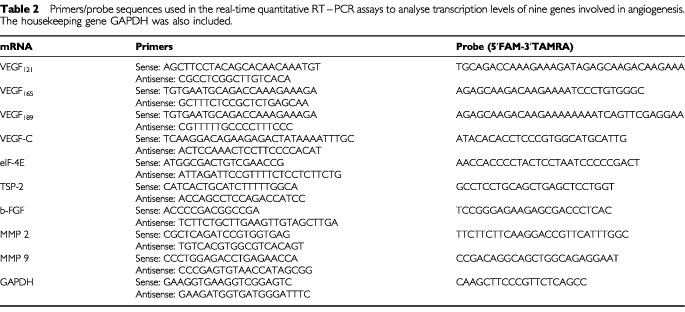
Primers/probe sequences used in the real-time quantitative RT–PCR assays to analyse transcription levels of nine genes involved in angiogenesis. The housekeeping gene GAPDH was also included

shows the primers and probe sequences for: VEGF_121_ (GenBank accession no: M27281), VEGF_165_ (GenBank accession no: M27281), VEGF_189_ (GenBank accession no: M27281), VEGF-C (GenBank accession no: X94216), eIF-4E (GenBank accession no: NM001968), TSP-2 (GenBank accession no: L12350), b-FGF (GenBank accession no: M27968), MMP-2 (GenBank accession no: M58552), MMP-9 (GenBank accession no: NM004994), and GAPDH (GenBank accession no: G04038).

### Single-step real-time quantitative RT–PCR

Real-time quantitative RT–PCR analysis was performed using the Taqman PCR Core Reagent Kit (PE Biosystems). Prior to reverse transcription, the RNA template was heated for 2 min at 50°C in the presence of 0.01 U l^−1^ uracil *N*-glycosylase. To prevent carry-over of contaminating DNA, the reaction was carried out in the presence of dUTP. Total RNA (100 ng) was reverse transcribed in a 25μl reaction mixture at 60°C for 30 min. After 5 min denaturation at 92°C, PCR was carried out for 40 cycles with denaturation at 92°C for 20 s and extension at 62°C for 60 s in the presence of the fluorescent oligonucleotides indicated above. RT–PCR analyses were carried out at least twice for each gene transcript to determine consistency of results, and GAPDH mRNA was assessed as internal control for RNA quality. Reactions were recorded and analysed using the ABI 7700 Prism Sequence detection system (Perkin-Elmer Applied Biosystems, Warrington, UK). Accurate quantitation was achieved through the generation of standard curves by serially diluting a known amount of cDNA from corresponding synthetic RNA and performing Taqman PCR on the series alongside patient samples. All gene transcript levels are expressed per μg of RNA.

### Preparation of cDNA

Two μg of DNase treated RNA was mixed with 2 μl (1 μg) of random hexamers in DEPC water, in a total volume of 14 μl. The mixture was heated at 75°C for 15 min and cooled on ice for 5 min. Subsequently, 5 μl of M-MLV RT reaction buffer, 5 μl of nucleotide pool and 1 μl of M-MLV RT enzyme was added. This mixture was left at room temperature for 10 min and incubated at 40°C for 50 min. Seventy five μl of DEPC water was added to make up a total volume of 100 μl.

### Real-time PCR for 18S rRNA

The housekeeping gene 18S rRNA was evaluated in all samples as internal control. In a total volume of 25 μl the following was added as reaction mixture: 12.5 μl of TaqMan Universal Master Mix (Applied Biosystems), 2.5 ul of human 18S rRNA Assay Reagents (Pre-developed; Applied Biosystems), 1 ul (100 ng) of cDNA and 9 μl of DEPC water. After 2 min at 50°C, denaturation was performed at 95°C for 10 min; PCR was carried out for 40 cycles with denaturation at 95°C for 15 s and annealing at 60°C for 1 min. The threshold cycle (Ct) for each sample was determined.

### Statistical Analysis

The Mann–Whitney *U-*test was used to compare the means of the gene expressions between normal and malignant tissues (SPSS software, Version 9.0 for Windows, SPSS, Inc, Chicago, IL, USA). We have used the Spearman's rank correlation to compare the co-expression patterns between the normal and the cancer group. A correlation with a *P*-value of <0.05 was considered to be significant. In addition, the z-test was performed to compare the Spearman correlation matrices between normal and malignant (MedCalc, Version 6.0 for Windows, 1998). The two compared correlation coefficients (*r*) are transformed into two z values by this formula:


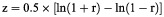


which almost follows a normal distribution of mean m, and variance 1/(*n*-3), with n the number of individuals in the sample. Then, for the statistical value D/SD_D_ a *P*-value is given:


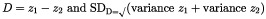


## RESULTS

### Transcript levels in normal and malignant cervical tissue

Real-time quantitative RT–PCR analysis was performed on mRNA transcripts of nine genes (VEGF_121_, VEGF_165_, VEGF_189_, VEGF-C, eIF-4E, b-FGF, TSP-2, MMP-2 and MMP-9) involved in angiogenesis and/or lymphangiogenesis as well as tumour cell invasion. Experiments were repeated at least twice for each gene. GAPDH was initially considered as an internal control. However, considerable variation in mRNA levels were found for this housekeeping gene. In contrast, all samples showed a threshold cycle between 17 and 18 for 18S rRNA, demonstrating its usefulness as internal control for quality of RNA.

Figures 1A, B, C

**Figure 1:**
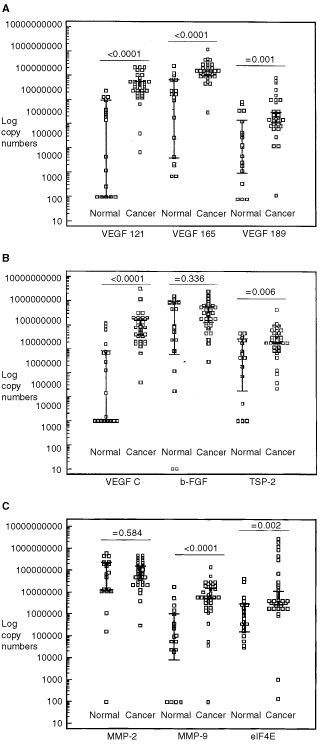
(**A**) Log mRNA copy numbers of the different VEGF-A splice variants in normal and malignant cervical tissue. (**B**) Log mRNA copy numbers of VEGF-C, b-FGF and Thrombospondin-2 in normal and malignant cervical tissue. (**C**) Log mRNA copy numbers of MMP-2, MMP-9 and eIF-4E in normal and malignant cervical tissue. Error bar: 95% Confidence Interval for the mean.

show the transcript levels in normal and malignant cervical tissues for the nine genes analysed. All tumour and normal samples expressed the VEGF-A isoforms in order of decreasing abundance, 165>121>189 (Figure 1A). In normal cervical tissue, a greater distribution (95% confidence interval (CI)) was observed for the mRNA copy numbers of the different VEGF-A splice variants compared to cervical cancer. This may be due in part to the influence of the hormonal cycle. The levels of the different VEGF-A splice variants were significantly higher in malignant compared to normal cervical tissues. There was an 11-fold increase in the mean copy number of VEGF_165_, a 23-fold increase in VEGF_121_ and a 13-fold increase in VEGF_189_ in malignant compared to normal tissues. The highest levels of VEGF_121_, VEGF_165_ and VEGF_189_ mRNA were found in cervical tumours with lympho-vascular space involvement in the primary tumour, which is known to be a poor prognostic factor (lympho-vascular space includes all small vessels, capillaries as well as lymphatic vessels, present in the primary tumour and the surrounding tissue). All cervical cancers expressed high levels of VEGF-C, and the levels were more than 130-fold higher than in normal cervical tissues (Figure 1B). The highest levels of VEGF-C mRNA were found in the lymph node-positive group. Of the 18 normal cervical tissues analysed, only 10 expressed >100 copies of VEGF-C mRNA/μg total RNA, suggesting that VEGF-C is not implicated in normal physiological processes in cervical tissue. Levels of b-FGF mRNA were similar in normal cervical tissue and early-stage cervical cancers. However, high levels of b-FGF mRNA were found in the five cervical cancers with advanced stage disease. The levels of TSP-2 and of eIF-4E mRNA were increased four- and seven-fold respectively in cervical cancer. No differences in the level of MMP-2 mRNA were found between normal and malignant cervical tissue. However, an approximately 80-fold increase in MMP-9 mRNA was observed in cervical cancers (Figure 1C).

Using the Mann–Whitney *U-*test the differences in the means between the two groups (normal *vs* cancer) are highly significant (*P*-values ⩽0.006), except for the genes b-FGF and MMP-2 (respective *P*-values: 0.336, 0.584) (Figure 1A, B, C).

### Gene co-expression patterns in normal and malignant cervical tissue

Tables 3A and B

**Table 3:**
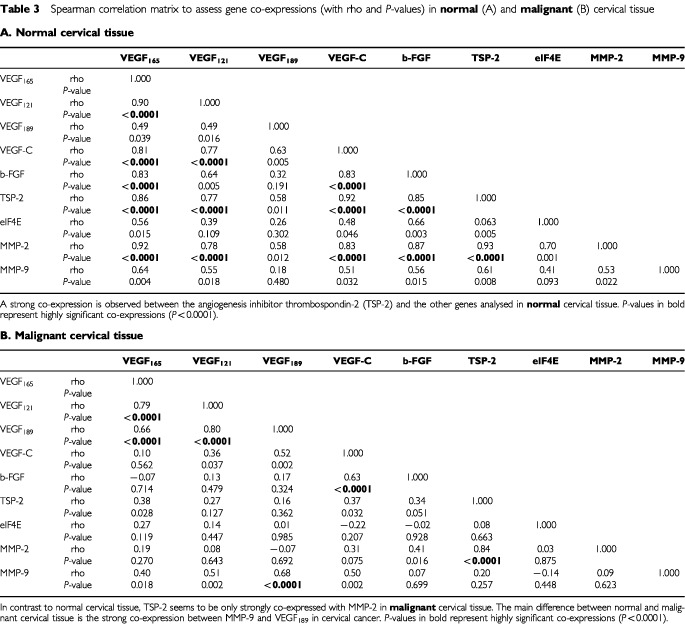
Spearman correlation matrix to assess gene co-expressions (with rho and *P*-values) in **normal** (A) and **malignant** (B) cervical tissue**A. Normal cervical tissue**

show the Spearman correlation matrices of gene co-expressions (with rho and *P*-values) in normal and malignant cervical tissue. In normal cervical tissue, a strong co-expression is observed between the angiogenesis inhibitor TSP-2 and the other genes analysed. Furthermore, MMP-2 is also strongly co-expressed with the other genes analysed in normal cervical tissue, except for MMP-9. In cervical cancer, TSP-2 seems to be strongly co-expressed only with MMP-2. In both normal and malignant cervical tissue, significant co-expressions were found between the different VEGF-A splice variants. In normal cervical tissue, VEGF-C is strongly co-expressed with all other genes analysed, except for MMP-9 and eIF4E. However, in cervical cancer, VEGF-C is strongly co-expressed with MMP-9, VEGF_189_ and b-FGF. In addition, MMP-9 is strongly co-expressed with VEGF_189_ in cervical cancer but not in normal cervical tissue.

To compare the co-expressions between the two groups (normal *vs* malignant), the z-statistic of Fischer was used on the Spearman correlation matrices (Table 4

**Table 4:**
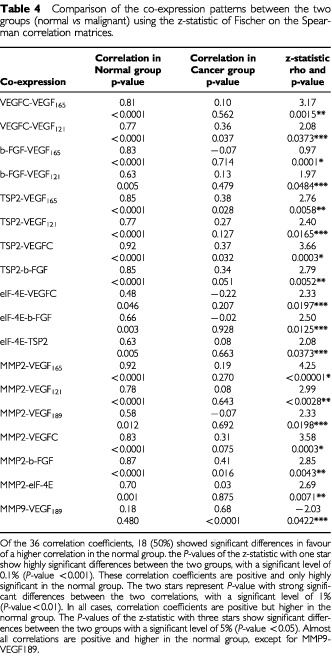
Comparison of the co-expression patterns between the two groups (normal *vs* malignant) using the z-statistic of Fischer on the Spearman correlation matrices

). Of the 36 correlation coefficients, 18 (50%) showed significant differences in favour of a higher correlation in the normal group, except for MMP-9/VEGF_189_.

Although the sample size was smaller for the normal group and the distribution (95% confidence interval) of quantitative gene expression levels was broader, we observed a highly consistent co-expression pattern in the normal cases. This could reflect the housekeeping mode in normal cases.

## Differences in gene (co-)expressions between recurrent and non-recurrent cervical cancers

Six patients (18%) with primary cervical cancer developed recurrent disease during a mean follow-up of 27 months. Three patients were initially diagnosed with early-stage disease, the other three patients had advanced disease at initial presentation. No significant differences in co-expression patterns were found between recurrent and non-recurrent cervical cancers. However, a significant difference was found in the mean of expression levels for TSP-2, between the recurrent and the non-recurrent group (*P*-value=0.038; Mann–Whitney *U*-test). The mean value of TSP-2 in non-recurrent cases was 4.9×10^5^ copies μg^−1^ RNA (s.d.: +/−7.7×10^5^), and in recurrent cases 1.8×10^5^ copies/μg RNA (SD: +/−3×10^5^). This suggests that TSP-2 might play a crucial role in progression of disease in cervical cancer.

## DISCUSSION

We show here that combined application of real-time quantitative RT–PCR with correlation matrices of gene expression data allows us to identify differences in gene co-expression patterns between normal and malignant cervical tissue.

*In vivo* and *in vitro* studies using semi-quantitative techniques have recently shown that tumours exhibiting high expression levels of vascular endothelial growth factor (VEGF) have a high metastatic potential (Potgens *et al*, 1996; Ohta *et al*, 1997). *In vivo* studies in cervical cancer have demonstrated a significant correlation between MVD and VEGF mRNA expression, with the highest levels in stage I and stage IV disease (Fujimoto *et al*, 1999; Kodama *et al*, 1999). VEGF_121_ and VEGF_165_ were found to be the two dominant VEGF-A subtypes with similar expression levels in normal cervix as well as in cervical cancer. VEGF_189_ was barely detectable. In our study, using a fully quantitative RT–PCR technique, mRNA levels of the different VEGF-A splice variants were significantly higher in malignant compared to normal cervical tissues, with a more than 10-fold increase in VEGF_121_, VEGF_165_, and VEGF_189_. Interestingly, the highest levels of VEGF_121_, VEGF_165_ and VEGF_189_ mRNA were found in cervical tumours histologically characterised by lympho-vascular space involvement, which is known to be a poor prognostic factor. In normal cervical tissue, a greater distribution was observed for the mRNA copy numbers of the different VEGF-A splice variants compared to cervical cancer. This may be due in part to the influence of the hormonal cycle, however, the co-expression patterns were stronger and more consistent than in cervical cancer.

We have shown that all tumour and normal samples expressed the VEGF-A isoforms, 165>121>189, in order of decreasing abundance. This order differs from normal and malignant breast tissue in which the VEGF_121_ isoform is most abundant (Scott *et al*, 1998). There is evidence that b-FGF mRNA levels are higher in advanced cervical cancers, regardless of histological type, suggesting that it may predominantly play a role in the later molecular events which regulate angiogenesis in this particular tumour (Fujimoto *et al*, 1997). Furthermore, it has been demonstrated that VEGF is essential for the initial but not continued growth of human breast carcinoma cells *in vivo*, and that other angiogenic factors, such as b-FGF, can substitute for VEGF during disease progression (Yoshiji *et al*, 1997). This is consistent with our findings that early-stage cervical cancers had no significant increase in b-FGF mRNA, whereas a significant increase was observed in advanced stage cancers. Elevated levels of eIF-4E have been associated with increased growth rates and cell transformation in HeLa cells (de Benedetti and Rhoads, 1990). It has been suggested that eIF-4E may regulate expression of genes including angiogenic factors such as VEGF and b-FGF as well as subsequent tumour growth (Kevil *et al*, 1996). In our study, there was a seven-fold increase in transcript level of eIF-4E in cervical cancer.

Clinical and pathological observations have revealed that lymph node involvement is one of the earliest features of metastatic disease, and this certainly holds true for cervical cancer. Blood-borne tumour cells seed out in the lymphatic hilum, and with an increasing number of tumour cells in the blood, the frequency of nodal involvement rises. This occurs particularly in patients with large volume disease and increased numbers of microvessels in the primary tumour. A second route is via lymphatic vessels, and this pathway may be of particular importance in early microdissemination to lymph nodes (Ohta *et al*, 2000; reviewed by van Trappen and Pepper, 2002). It has recently been demonstrated that primary solid tumours expressing vascular endothelial growth factors -C and -D (VEGF-C and -D), induce lymphangiogenesis *de novo*, thereby providing a direct conduit for tumour cell dissemination to lymph nodes (Mandriota *et al*, 2001; Skobe *et al*, 2001; Stacker *et al*, 2001). In prostatic carcinoma, the expression of VEGF-C mRNA in the primary tumour is significantly higher in lymph node-positive patients compared to lymph node-negative patients (Tsurusaki *et al*, 1999).

Our results have revealed that VEGF-C transcript levels are more than 130-fold higher in cervical cancer compared to normal cervical tissues. Using real-time quantitative RT–PCR we found the highest VEGF-C mRNA levels in the lymph node-positive cervical cancers. Although VEGF-C is the principal lymphangiogenic growth factor described to date, it also has angiogenic properties under certain circumstances, including tumorigenesis (Olofsson *et al*, 1999). This implies that VEGF-C may serve to increase both tumour vascularisation and lymphangiogenesis, thereby contributing to the increase in tumour growth and the formation of metastases.

Proteolytically-mediated extracellular matrix (ECM) degradation is crucial for angiogenesis as well as for the detachment of malignant cells from the primary tumour and migration through the surrounding stroma (Pepper, 2001). Matrix metalloproteinases (MMPs) have been heavily implicated in these processes, and are indispensable for the degradation of native triple-helical collagen, a major component of ECMs. Two of the best studied MMP's in angiogenesis and cancer are MMP-2 and MMP-9 (gelatinase-A and -B), which amongst other molecules degrade collagen IV, one of the major components of the basement membrane. MMP-2 and MMP-9 play an important role in triggering the angiogenic switch in null mice (reviewed by Pepper, 2001). In addition, enhanced mRNA and protein expression of both enzymes has been reported in breast, colon and pancreatic cancer (Campo *et al*, 1992). In our study, no differences in MMP-2 were found between normal and malignant cervical tissue at the mRNA level. However, an approximately 80-fold increase was observed for MMP-9 mRNA in cervical cancers. The fact that we observed no increase in MMP-2 and a marked increase in MMP-9, provides no information about the status of MMP activation, which from a mechanistic point of view may be more relevant. Nonetheless, our findings have revealed a clear relationship between increased MMP-9 mRNA and cervical cancer, increasing the probability that MMP-9 plays a key role in the progression of early-stage cervical cancer.

In normal cervical tissue, gene co-expression pattern analysis revealed a strong co-expression between TSP-2, MMP-2 and the other genes analysed, except for MMP-9. In cervical cancer, TSP-2 appears only to be co-expressed with MMP-2. The lymphangiogenic factor VEGF-C is strongly co-expressed with MMP-9, VEGF_189_ and b-FGF in cervical cancer. In addition, MMP-9 is strongly co-expressed with VEGF_189_ in cervical cancer but not in normal cervical tissue. Comparison of the co-expression patterns between the two groups revealed that almost all correlations are positive and stronger in the normal group, except for the co-expression MMP-9/VEGF_189_. Recently, *in vivo* studies have shown that colon cancer metastasis results from an alteration in the balance between the angiogenesis inhibitor thrombospondin-2 and the angiogenic growth factor VEGF_189_ (Tokunaga *et al*, 1998). We found a significant difference in the mean of expression levels for thrombospondin-2 between recurrent and non-recurrent cancer cases, with the lowest levels in the recurrent cases. VEGF_189_ has been shown to correlate with poor prognosis in colon and lung cancer (Oshika *et al*, 1998; Tokunaga *et al*, 1998), and VEGF-C may facilitate lymphatic spread (Mandriota *et al*, 2001; Skobe *et al*, 2001; Stacker *et al*, 2001). In our study, both VEGF_189_ and VEGF-C appear to be strongly co-expressed with MMP-9 in cervical cancer but not in normal cervical tissue.

In this report, we described the combined application of fully quantitative gene expression data with correlation matrices. This approach revealed different co-expression patterns between normal and malignant cervical tissue. This combined molecular-statistical model could be applied to several other cancers or other sets of genes.

## References

[bib1] BergersGBrekkenRMcMahonGVuTHItohTTamakiKTanzawaKThorpePItoharaSWerbZHanahanD2000 Matrix metalloproteinase-9 triggers the angiogenic switch during carcinogenesis Nat Cell Biol 2 737744 11025665 10.1038/35036374 PMC2852586

[bib2] CampoEMerinoMJLiottaLNeumannRStetler-StevensonW1992 Distribution of the 72-kd type IV collagenase in nonneoplastic and neoplastic thyroid tissue Hum Pathol 23 13951401 1468777 10.1016/0046-8177(92)90060-g

[bib3] CarmelietPJainR2000 Angiogenesis in cancer and other diseases Nature 407 249257 11001068 10.1038/35025220

[bib4] ChomczynskiPSacchiN1987 Single-step method of RNA isolation by acid guanidinium thiocyanate-phenol-chloroform extraction Anal Biochem 162 156159 2440339 10.1006/abio.1987.9999

[bib5] De BenedettiAHarrisAL1999 eIF4E expression in tumors: its possible role in progression of malignancies Int J Biochem Cell Biol 31 5972 10216944 10.1016/s1357-2725(98)00132-0

[bib6] De BenedettiARhoadsRE1990 Overexpression of eukaryotic protein synthesis initiation factor 4E in HeLa cells results in aberrant growth and morphology Proc Natl Acad Sci USA 87 82128216 2122455 10.1073/pnas.87.21.8212 PMC54925

[bib7] DugganDJBittnerMChenYMeltzerPTrentJM1999 Expression profiling using cDNA microarrays Nature Genetics 21 1014 9915494 10.1038/4434

[bib8] EisenMBSpellmanPTBrownPOBotsteinD1998 Cluster analysis and display of genome-wide expression patterns Proc Natl Acad Sci USA 95 1486314868 9843981 10.1073/pnas.95.25.14863 PMC24541

[bib9] FerraraNHouckKAJakemanLBWinerJLeungDW1991 The vascular endothelial growth factor family of polypeptides J Cell Biochem 47 211218 1791185 10.1002/jcb.240470305

[bib10] FolkmanJ2000 Incipient angiogenesis J Natl Cancer Inst 92 9495 10639502 10.1093/jnci/92.2.94

[bib11] FolkmanJ1971 Tumor angiogenesis: therapeutic implications N Engl J Med 285 11821186 4938153 10.1056/NEJM197111182852108

[bib12] FujimotoJIchigoSHiroseRSakaguchiHTamayaT1997 Expression of basic fibroblast growth factor and its mRNA in advanced uterine cervical cancers Cancer Lett 111 2126 9022124 10.1016/s0304-3835(96)04485-0

[bib13] FujimotoJSakaguchiHHiroseRIchigoSTamayaT1999 Expression of vascular endothelial growth factor (VEGF) and its mRNA in uterine cervical cancers Br J Cancer 80 827833 10360662 10.1038/sj.bjc.6690428 PMC2362279

[bib14] HanahanDFolkmanJ1996 Patterns and emerging mechanisms of the angiogenic switch during tumorigenesis Cell 86 353364 8756718 10.1016/s0092-8674(00)80108-7

[bib15] HeidCAStevensJLivakKJWilliamsPM1996 Real time quantitative PCR Genome Res 6 986994 8908518 10.1101/gr.6.10.986

[bib16] HouckKAFerraraNWinerJCachianesGLiBLeungDW1991 The vascular endothelial growth factor family: identification of a fourth molecular species and characterization of alternative splicing of RNA Mol Endocrinol 5 18061814 1791831 10.1210/mend-5-12-1806

[bib17] JeltschMKaipainenAJoukovVMengXLaksoMRauvalaHSwartzMFukumuraDJainRKAlitaloK1997 Hyperplasia of lypmatic vessels in VEGF-C transgenic mice Science 276 14231425 9162011 10.1126/science.276.5317.1423

[bib18] KevilCGDe BenedettiAPayneDKCoeLLLarouxFSAlexanderJS1996 Translational regulation of vascular permeability factor by eukaryotic initiation factor 4E: implications for tumor angiogenesis Int J Cancer 65 785790 8631593 10.1002/(SICI)1097-0215(19960315)65:6<785::AID-IJC14>3.0.CO;2-3

[bib19] KodamaJSekiNTokumoKHongoAMiyagiYYoshinouchiMOkudaHKudoT1999 Vascular endothelial growth factor is implicated in early invasion in cervical cancer Eur J Cancer 35 485489 10448304 10.1016/s0959-8049(98)00410-9

[bib20] LanderES1999 Array of hope Nature Genetics 21 34 9915492 10.1038/4427

[bib21] LiottaLPetricoinE2000 Molecular profiling of human cancer Nature Reviews Genetics 1 4856 10.1038/35049567 11262874

[bib22] LymboussakiAPartanenTAOlofssonBThomas-CrusellsJFletcherCDde WaalRMKaipainenAAlitaloK1998 Expression of the vascular endothelial growth factor C receptor VEGFR-3 in lymphatic endothelium of the skin and in vascular tumors Am J Pathol 153 395403 9708800 10.1016/S0002-9440(10)65583-6 PMC1852985

[bib23] MandriotaSJJussilaLJeltschMCompagniABaetensDPrevoRBanerjiSHuarteJMontesanoRJacksonDGOrciLAlitaloKChristoforiGPepperMS2001 Vascular endothelial growth factor-C mediated lymphangiogenesis promotes tumour metastasis EMBO 20 672682 10.1093/emboj/20.4.672 PMC145430 11179212

[bib24] OhtaYNozawaHTanakaYOdaMWatanabeY2000 (Apr) Increased vascular endothelial growth factor and vascular endothelial growth factor-c and decreased nm23 expression associated with microdissemination in the lymph nodes in stage I non-small cell lung cancer J Thorac Cardiovasc Surg 119 804813 10733773 10.1016/S0022-5223(00)70017-1

[bib25] OhtaYWatanabeYMurakamiSOdaMHayashiYNonomuraAEndoYSasakiT1997 Vascular endothelial growth factor and lymph node metastasis in primary lung cancer Br J Cancer 76 10411045 9376264 10.1038/bjc.1997.505 PMC2228101

[bib26] OlofssonBJeltschMErikssonUAlitaloK1999 Current biology of VEGF-B and VEGF-C Curr Opin Biotechnol 10 528535 10600689 10.1016/s0958-1669(99)00024-5

[bib27] O'ReillyMSBoehmTShingYFukaiNVasiosGLaneWSFlynnEBirkheadJROlsenBRFolkmanJ1997 Endostatin: an endogenous inhibitor of angiogenesis and tumor growth Cell 88 277285 9008168 10.1016/s0092-8674(00)81848-6

[bib28] O'ReillyMSHolmgrenLShingYChenCRosenthalRAMosesMLaneWSCaoYSageEHFolkmanJ1994 Angiostatin: a novel angiogenesis inhibitor that mediates the suppression of metastases by a Lewis Lung Carcinoma Cell 79 315328 7525077 10.1016/0092-8674(94)90200-3

[bib29] OshikaYNakamuraMTokunagaTOzekiYFukushimaYHatanakaHAbeYYamazakiHKijimaHTamaokiNUeyamaY1998 Expression of cell-associated isoform of vascular endothelial growth factor 189 and its prognostic relevance in non-small cell lung cancer Int J Oncol 12 541544 9472090 10.3892/ijo.12.3.541

[bib30] PepperMS2001 Role of the matrix metalloproteinase and plasminogen activator-plasmin systems in angiogenesis Arterioscler Thromb Vasc Biol 21 11041117 11451738 10.1161/hq0701.093685

[bib31] PotgensAJvan AltenaMCLubsenNHRuiterDJde WaalRM1996 Analysis of the tumor vasculature and metastatic behavior of xenografts of human melanoma cell lines transfected with vascular permeability factor Am J Pathol 148 12031217 8644861 PMC1861532

[bib32] SchenaMShalonDDavisRWBrownPO1995 Quantitative monitoring of gene expression patterns with a complementary DNA microarray Science 270 467470 7569999 10.1126/science.270.5235.467

[bib33] ScottPASmithKPoulsomRDe BenedettiABicknellRHarrisAL1998 Differential expression of vascular endothelial growth factor mRNA vs protein isoform expression in human breast cancer and relationship to eIF-4E Br J Cancer 77 21202128 9649123 10.1038/bjc.1998.356 PMC2150428

[bib34] SkobeMHawighorstTJacksonDGPrevoRJanesLVelascoPRiccardiLAlitaloKClaffeyKDetmarM2001 Induction of tumor lymphangiogenesis by VEGF-C promotes breast cancer metastasis Nat Med 7 192198 11175850 10.1038/84643

[bib35] StackerSACaesarCBaldwinMEThorntonGEWilliamsRAPrevoRJacksonDGNishikawaSKuboHAchenMG2001 VEGF-D promotes the metastatic spread of tumor cells via the lymphatics Nat Med 7 186191 11175849 10.1038/84635

[bib36] TokumoKKodamaJSekiNNakanishiYMiyagiYKamimuraSYoshinouchiMOkudaHKudoT1998 Different angiogenic pathways in human cervical cancers Gynecol Oncol 68 3844 9454658 10.1006/gyno.1997.4876

[bib37] TokunagaTOshikaYAbeYOzekiYSadahiroSKijimaHTsuchidaTYamazakiHUeyamaYTamaokiNNakamuraM1998 Vascular endothelial growth factor (VEGF) mRNA isoform expression pattern is correlated with liver metastasis and poor prognosis in colon cancer Br J Cancer 77 9981002 9528847 10.1038/bjc.1998.164 PMC2150098

[bib38] TsurusakiTKandaSSakaiHKanetakeHSaitoYAlitaloKKojiT1999 Vascular endothelial growth factor-C expression in human prostatic carcinoma and its relationship to lymph node metastasis Br J Cancer 80 309313 10390013 10.1038/sj.bjc.6690356 PMC2362987

[bib39] Van TrappenPOGyselmanVGLoweDGRyanAOramDHBoszePWeekesARShepherdJHDorudiSBustinSAJacobsIJ2001 Molecular quantification and mapping of lymph node micrometastases in cervical cancer Lancet 357 1520 11197354 10.1016/S0140-6736(00)03566-2

[bib40] Van TrappenPOPepperMS2002 Lymphatic dissemination of tumour cells and the formation of tumour micrometastases Lancet Oncol 3 4452 11905605 10.1016/s1470-2045(01)00621-0

[bib41] VeikkolaTAlitaloK1999 VEGFs, receptors and angiogenesis Semin Cancer Biology 9 211220 10.1006/scbi.1998.0091 10343072

[bib42] VolpertOVTolsmaSSPellerinSFeigeJJChenHMosherDFBouckN1995 Inhibition of angiogenesis by thrombospondin-2 Biochem Biophys Res Commun 217 326332 8526929 10.1006/bbrc.1995.2780

[bib43] YancopoulosGDDavisSGaleNWRudgeJSWiegandSJHolashJ2000 Vascular-specific growth factors and blood vessel formation Nature 407 242248 11001067 10.1038/35025215

[bib44] YoshijiHHarrisSRThorgeirssonUP1997 Vascular endothelial growth factor is essential for initial but not continued in vivo growth of human breast carcinoma cells Cancer Res 57 39243928 9307273

